# Learning organizations, internal marketing, and organizational commitment in hospitals

**DOI:** 10.1186/1472-6963-14-152

**Published:** 2014-04-04

**Authors:** Yafang Tsai

**Affiliations:** 1 Department of Health Policy and Management, Chung Shan Medical University, No.110, Sec., Jianguo N. Rd., Taichung City, 40201, Taiwan; 2 Department of Medical Management, Chung Shan Medical University Hospital, No.110, Sec, Jianguo N. Rd, Taichung City, 40201, Taiwan

**Keywords:** Nursing management, Learning organization, Internal marketing, Organizational commitment

## Abstract

**Background:**

Knowledge capital is becoming more important to healthcare establishments, especially for hospitals that are facing changing societal and industrial patterns. Hospital staff must engage in a process of continual learning to improve their healthcare skills and provide a superior service to their patients. Internal marketing helps hospital administrators to improve the quality of service provided by nursing staff to their patients and allows hospitals to build a learning culture and enhance the organizational commitment of its nursing staff. Our empirical study provides nursing managers with a tool to allow them to initiate a change in the attitudes of nurses towards work, by constructing a new ‘learning organization’ and using effective internal marketing.

**Methods:**

A cross-sectional design was employed. Two hundred questionnaires were distributed to nurses working in either a medical centre or a regional hospital in Taichung City, Taiwan, and 114 valid questionnaires were returned (response rate: 57%). The entire process of distribution and returns was completed between 1 October and 31 October 2009. Hypothesis testing was conducted using structural equation modelling.

**Results:**

A significant positive correlation was found between the existence of a ‘learning organization’, internal marketing, and organizational commitment. Internal marketing was a mediator between creating a learning organization and organizational commitment.

**Conclusion:**

Nursing managers may be able to apply the creation of a learning organization to strategies that can strengthen employee organizational commitment. Further, when promoting the creation of a learning organization, managers can coordinate their internal marketing practices to enhance the organizational commitment of nurses.

## Background

For several years, Taiwan’s Bureau of National Health Insurance (NHI) has focussed on reducing its financial deficit. Consequently, in common with their counterparts abroad, hospital managers in Taiwan have faced the challenge of having to control costs while maintaining service quality. Knowledge capital is becoming more important to healthcare establishments, especially for hospitals facing changing societal and industrial patterns [1]. Nursing staff must engage in a process of continual learning to improve their care skills and provide a superior service to their patients. Hospital administrators can enhance creativity and efficiency through individual learning, satisfy the demands inherent within the duties of healthcare staff, and improve organizational commitment by promoting the notion of being a ‘learning organization’ [2].

Healthcare organizations are highly knowledge-intensive institutions that require continual learning to improve their capabilities. Establishing a culture of learning is an important issue for them [3]. Jeong et al. [2] also suggested that future studies should explore the effect of other variables on the concept of the ‘learning organization’.

Globally, nursing leaders face challenges of manpower shortages and high turnover rates [4]. Organizational commitment is a predictor of turnover intention in nursing staff [5], and nursing managers must therefore find effective management approaches capable of influencing organizational commitment. Internal marketing is a useful management tool for this [6].

### Purpose and importance of the present study

One of the main purposes of nursing is not only to provide patients with excellent care, but also to ensure they are satisfied with that care. The existing literature indicates that if nursing leaders can alter the personal work attitudes of nurses, this can in turn enhance the level of service provided to patients. The organizational commitment and job satisfaction of individual nurses influence service quality [6]. This raises the question of how changes in the work attitudes of nurses can be accomplished. Our empirical study identifies a tool for nursing managers which would allow them to initiate a change in the work attitude of nurses, by putting in place the principles of a learning organization and conducting internal marketing.

### Learning organizations

An implication of the promotion of patient-centred healthcare and the advancement of healthcare technology is that nursing staff must continually upgrade their competence through ongoing learning, to both satisfy patient needs and ensure consistent quality of service provision. These factors have encouraged healthcare establishments to declare themselves ‘learning organizations’ to enhance the continual education of healthcare staff, thereby improving problem-solving capacity and the provision of a timely response to patients’ needs [7].

The learning organization has been defined as an ideal organizational vision which could help organizations to cope with and even lead environmental change by reinforcing learning activities [2]. To ensure that nurses are equipped with the necessary ability to adapt to technological and environmental change, nursing managers should provide staff with continual education and training. This could ensure that nurses have the ability to provide patients with a consistent quality of nursing care.

### Internal marketing

Internal marketing is a human resource management tool used by organizations to successfully educate, train, and motivate employees to provide better services to customers [8]. In healthcare, internal marketing is primarily concerned with the ways in which the organization’s management develops educational training, explicitly communicates organizational perspectives, and creates reward systems that improve the nursing staff’s ability and satisfaction with their work [6]. Studying hospital nurses, Tsai and Wu [6] found that the managerial activities used in executing internal marketing included human resource management activities (e.g. hospitals designed a policy of rewarding nursing effort at work and reaching organizational performance targets, nursing leaders satisfied the work needs of nurses, and nursing managers provided educational training opportunities for nurses) and vision and development (e.g. nursing managers provided nurses with a vision they could trust and took into consideration the development and improvement of nursing skills).

### Organizational commitment

Organizational commitment is the collection of feelings and beliefs that managers have about their organization as a whole [9]. Allen and Meyer [10] identified three types of organizational commitment: affective (individual emotional attachment to, identification with and involvement in a particular organization), continuance (an employee’s awareness of the cost of leaving an organization), and normative (an individual’s sense that they were obliged to remain in an organization). Allen and Meyer’s [10] definition is commonly used in nursing studies [11] including this one.

### Theoretical relationships between the concepts of learning organizations, internal marketing, and organizational commitment

A primary goal for organizations attempting to achieve a learning organization is to mould an organizational culture of learning [12]. To this end, Watkins and Ellinger [13] suggested that hospital administrators should encourage dialogue, interaction, and knowledge exchange between members of different departments, including nursing staff. Another study suggested that creating a learning organization was made easier by establishing internal communication channels to announce the policies, vision, and targets of the organization [14]. Use of internal marketing encourages nursing management to transmit the visions and goals of their hospital [15]. It also allows nursing staff to understand the overall aims and mission. Internal marketing emphasizes that the organization needs to allow staff to strengthen their abilities through training, and nurture service-oriented behaviour by clarifying the organizational vision [16]. Our first hypothesis is therefore as follows:

Hypothesis 1- Creating a learning organization influences internal marketing.

Organizational commitment indicates the degree to which individuals are attached to an organization and how they identify with its task goals [10]. Healthcare organizations can use internal marketing to enhance the organizational commitment of nursing staff [17]. We have therefore developed our second hypothesis as:

Hypothesis 2- Internal marketing influences organizational commitment.

In addition to encouraging ongoing learning among nursing staff, learning organizations place emphasis on the ways in which leaders can empower their employees [18]. When nurses are able to resolve a patient’s problem in a timely manner, patients tend to be satisfied with their care. Patient satisfaction is a reflection of the commitment and professionalism displayed by nurses and realised in their performance outcomes. Fostering the professional commitment of nurses is helpful in encouraging commitment to an organization [19]. Our third hypothesis is therefore:

Hypothesis 3- Creating a learning organization influences organizational commitment.

An organization that can continue to provide education and training to its nurses can ensure the long-term, stable provision of quality care. Providing quality care can also result in an increase in recognition of the nursing profession by patients, thus reducing the frustration that some nurses feel with the healthcare process, which can negatively affect their organizational commitment. Jeong et al. [2] empirically showed that when nursing leaders practise learning organization principles, the organizational commitment of nurses can change. Additionally, Tsai and Wu [6] found that the practice of internal marketing affects the organizational commitment of nurses. Therefore, when nursing managers practise learning organization principles, this will affect the organization’s internal marketing, and in turn, the organizational commitment of nursing staff. Consequently, we propose our fourth hypothesis:

Hypothesis 4 - Internal marketing is a mediator between creating a learning organization and organizational commitment.

## Methods

### Development of the instrument

The instrument contained the following subscales: learning organization (LO; 20 items), internal marketing (IM; 14 items), and organizational commitment (OC; 15 items). Given the latent character of the variables considered in this study, a five-point Likert scale was used for responses (1 = *strongly disagree* and 5 = *strongly agree*).

Items were initially developed by consulting the relevant literature. Three nursing supervisors (from three Taiwan medical centres) were invited to perform an expert validation of the questionnaire, after which it was revised further. A pilot study was then conducted by administering the questionnaire to 50 nurses in a medical centre. The results indicated that all of the scales and subscales had satisfactory reliability (Cronbach’s α > 0.70) [20].

### Design, sampling, and participants

This empirical study had a cross-sectional design. The survey was based on voluntary participation. All responses were anonymous, and the respondents and the researchers were not acquainted with each other. The researchers invited the respondents to participate in this research by distributing the questionnaires. At the same time, the respondents were informed that all information was provided on a voluntary basis and would be used for research purposes only. Because names were not required on the data collection forms, privacy and anonymity were ensured. To guarantee the respondents’ privacy, the questionnaires were sealed after they were retrieved to keep their contents secure and anonymous. If the participants did not agree to provide the information, they did not return the questionnaire. This research was approved by the Institutional Review Board of Chung Shan Medical University Hospital. The clinical trial code was CS14013.

Questionnaires were distributed to 200 nurses working in either a medical centre or a regional hospital in Taichung City, Taiwan, and 114 valid questionnaires were returned (response rate: 57%). The participants of this research were all adult employees. The entire process of distribution and return was completed between 1 and 31 October 2009.

The majority of subjects were 31–40 years old (50.0%) and were female (86.8%). Of these, 25.4% had been working for 7–9 years, and 54.4% had a university education (Table 1).

**Table 1:** Participant demographics

**Variables**	**Number**	**%**
Gender (*n* = 114)		
Female	99	86.8
Male	15	13.2
Tenure (*n* = 114)		
<1 years	11	9.6
1-3 years	21	18.4
4-6 years	26	22.8
7-9 years	29	25.4
10-15 years	14	12.4
>15 years	11	9.6
Missing data	2	1.8
Educational level (*n* = 114)		
College	36	31.6
University	62	54.4
Postgraduate	13	11.4
Missing data	3	2.6
Position in hospital (*n* = 114)		
Nurse	97	85.1
Nursing supervisor	14	12.3
Missing data	3	2.6
Hospital level (*n* = 114)		
Medical center	70	61.4
Regional hospital	44	38.6
Age (years, *n* = 114)		
20-30	33	28.9
31-40	57	50.0
41-50	20	17.5
51-60	2	1.8
Missing data	2	1.8

### Data analysis

Descriptive statistics (including the mean (M) and standard deviation (SD) of each item and construct) were generated. An exploratory factor analysis (EFA) was performed to explore the factor structure, using Cronbach’s α to evaluate the internal consistency of factors. The data were analysed using structural equation modelling (SEM), with latent variables. The model parameters were estimated via maximum likelihood (ML) estimation, using AMOS 7.0. Indirect effects were tested using a bootstrap framework, based on Shrout and Bolger’s [20] techniques. We used 5,000 bootstrap samples (re-sampled from the original dataset) to mitigate biased standard errors and achieve a bias-corrected 95% confidence interval (BC 95% CI).

Several indices of model fitness, such as the chi-square ratios (χ^2^/df), adjusted goodness of fix index (AGFI), standardized root-mean-squared residual (SRMR), comparative fit index (CFI), and root-mean-square error of approximation (RMSEA) were used to evaluate the overall fit of the model. A χ^2^/*df* less than 3, an AGFI greater than 0.90, a CFI greater than 0.95, an SRMR less than 0.08, and an RMSEA less than 0.08 were regarded as indicators of a good model fit.

### Validity and reliability of the instrument

For the formal study, 114 valid questionnaires were collected. Additional file 1: Table S1, Additional file 2: Table S2 and Additional file 3: Table S3 summarize the descriptive statistics, EFA, and internal consistency (Cronbach’s α) for all constructs and variables. A corrected item-total correlation was used to evaluate the quality of items prior to performing the EFA [21].

#### Factor analysis

A principal component analysis was used to extract the major contributing factors and a Varimax rotation (orthogonal) was performed to identify the common factors. Factors with eigenvalues greater than 1 were also extracted. A factor loading greater than 0.40 was regarded as ‘practically significant’ in accordance with Hair et al. [22]. The Cronbach’s α for all of the sub-constructs exceeded 0.65, which is considered acceptable for internal consistency [23].

#### Convergent validity

A parameter (λ) estimate was calculated for each latent and observational variable, to determine statistical significance and evaluate convergent validity. Except for one sub-construct of organizational commitment (Continuance commitment), all *t* values exceeded 2 and all standardized coefficients were larger than 0.45 (yielding an *R*^2^ larger than 0.20). This indicated an acceptable convergent validity (Table 2). Additionally, the composite reliability of learning organization (LO) and internal marketing (IM) was greater than 0.70 and the average variance extraction (AVE) for both constructs exceeded 0.50. This indicated that the observational variables were derived from the latent variables. However, the indices of convergent reliability (CR) and AVE for organizational commitment did not meet the criterion.

**Table 2:** Results of measurement model of SEM analysis (model 1 with path c’)

**Construct/Item**	**SFL**	***t*-Value**	**SMC**	**CR**	**AVE**
**Learning Organization (LO)**				0.85	0.66
Learning	0.85	-	0.72		
Communication	0.84	10.27^***^	0.70		
Information	0.74	8.82^***^	0.55		
**Internet Marketing (IM)**				0.78	0.63
Vision & development	0.83	-	0.68		
Human resource management	0.77	8.59^***^	0.59		
**Organizational Commitment (OC)**				0.57	0.34
Affective commitment	0.66	-	0.43		
Normative commitment	0.72	6.54^***^	0.52		
Continuance commitment	0.23	2.26^*^	0.05		

Note: *SFL* standardized factor loading, *SMC* squared multiple correlation, *CR* Construct reliability, *AVE* Average variance explained.

*p <0.05, ***p <0.001.

## Results

### Descriptive statistics and correlation analysis

The means, standard deviations, and bivariate correlations for all the observational variables are shown in Table 3. The mean values ranged from 3.46 to 3.64 for learning organization, 3.48 to 3.49 for internal marketing, and 3.21 to 3.46 for organizational commitment.

**Table 3:** Descriptive statistics and correlations for all study variables (n = 114)

**No.**	**Variables**	***M***	***SD***	**1**	**2**	**3**	**4**	**5**	**6**	**7**	**8**	**9**	**10**
1	**Learning Organization (LO)**	3.58	0.50	-									
2	Learning	3.64	0.52	0.92^***^	-								
3	Communication	3.46	0.61	0.88^***^	0.68^***^	-							
4	Information	3.62	0.58	0.82^***^	0.62^***^	0.69^***^	-						
5	**Internal Marketing (IM)**	3.48	0.50	0.71^***^	0.65^***^	0.66^***^	0.55^***^	-					
6	Vision & development	3.48	0.53	0.68^***^	0.63^***^	0.62^***^	0.51^***^	0.92^***^	-				
7	Human resource management	3.49	0.59	0.60^***^	0.53^***^	0.56^***^	0.48^***^	0.88^***^	0.63^***^	-			
8	**Organization Commitment (OC)**	3.37	0.41	0.65^***^	0.68^***^	0.54^***^	0.42^***^	0.68^***^	0.60^***^	0.63^***^	-		
9	Affective commitment	3.39	0.55	0.55^***^	0.56^***^	0.45^***^	0.40^***^	0.58^***^	0.52^***^	0.52^***^	0.78^***^	-	
10	Normative commitment	3.46	0.56	0.61^***^	0.64^***^	0.50^***^	0.38^***^	0.62^***^	0.58^***^	0.53^***^	0.81^***^	0.48^***^	-
11	Continuance commitment	3.21	0.65	0.19^*^	0.22^*^	0.18	0.07	0.21^*^	0.12	0.27^**^	0.52^***^	0.02	0.26^**^

Note: Number 1 indicates the variables of Learning Organization; Number 2 indicates the Learning dimension of LO; Number 3 indicates the Communication dimension of LO; Number 4 indicates the Information construct of LO; Number 5 indicates the variables of Internal Marketing; Number 6 indicates the Vision and Development constructs of IM; Number 7 indicates the Human Resource Management construct of IM; Number 8 indicates the variables of Organizational Commitment; Number 9 indicates the Affective Commitment construct of OC; Number 10 indicates the Normative Commitment construct of OC; Number 11 indicates the Continuance Commitment construct of OC.

Note: *p < 0.05, **p < 0.01, ***p < 0.001.

The three constructs of Learning Organization (Learning, Communication, and Information) and the two constructs of Internal Marketing (Vision and Development, and Human Resource Management) were all significantly correlated, almost all with a correlation coefficient value >0.5.

The three constructs of Learning Organization (Learning, Communication, and Information) and the two constructs of Organizational Commitment (Affective Commitment and Normative Commitment) were all significantly correlated. However, when compared with those of Learning Organization and Internal Marketing, the correlation coefficient values were lower, between 0.19 and 0.22. The Communication and Information constructs of Learning Organization and the Continuance Commitment construct of Organizational Commitment did not exhibit significant correlation. This is probably because respondents with a low continuance commitment had low identification with their employing hospital. Further information from hospital managers does not improve this identification.

The Vision and Development construct of Internal Marketing and the Continuance Commitment also did not show statistical significance. This may be because hospital vision and development are seen as a concern of hospital management, not frontline staff. Furthermore, when respondents have a low identification with the hospital, they do not identify with the vision and future development of the hospital.

### The relationship between learning organization, internal marketing and organizational commitment (direct effects in the structural model)

The path coefficient ‘a’ from learning organization to internal marketing was statistically significant (βs = 0.87, *t* = 8.40, *p* < 0.001) (Table 4). This indicates that higher levels of perception of the existence of a learning organization were associated with increased internal marketing. We also found a positive association between internal marketing and organization commitment (path coefficient ‘b’; βs = 0.81, *t* = 2.52, *p* < 0.05). However, the association between the existence of a learning organization and organizational commitment was not significant (the path coefficient ‘c’ , when controlling the effect of internal marketing), which implies the possibility of ‘full mediation’ [24].

**Table 4:** Standard ML and bootstrapping methods for the estimation of structural model

	**ML estimates**				**Bootstrapping estimates of β**				**BC 95% CI of β**	
**Effect/Path way**	**β**	***SE***	**βs**	***t*-value**	***SE***	***SE*-*SE***	**Bias**	***SE*-Bias**	**Lower**	**Upper**
*Direct effect*										
a. LO → IM	0.86	0.102	0.87	8.40^***^	0.116	0.001	0.001	0.002	0.66	1.11
b. IM → OC	0.67	0.264	0.81	2.52^*^	0.740	0.007	0.152	0.010	0.03	2.13
c’. LO → OC	0.15	0.238	0.19	0.64	0.725	0.007	−0.147	0.010	−1.18	0.78
*Indirect effect*										
a × b	0.57	0.236^‡^	0.71	2.41^*‡^	0.728				0.09	2.42

Note: Based on 5,000 bootstrap samples; *ML* maximum likelihood, *BC 95% CI* biased corrected confidence intervals (95%), *β* unstandardized path coefficient, *βs* standardized path coefficient, *SE* standard error; ^‡^computed according to Sobel (1982) formula of indirect effect.

*p < 0.05, ***p < 0.001.

### The mediation effect of internal marketing on the relationship between learning organization and organizational commitment (indirect effect in the structural model)

The indirect effect of internal marketing with a bootstrapped standard error composed of 5,000 re-samples, a bias-corrected 95% CI and a Sobel test was calculated [23]. The indirect effect was significant (Sobel *Z* = 2.41, BC 95% CI of *B* = 0.09–2.42), indicating a mediation effect precipitated by internal marketing. As the direct path was not significant (βs = 0.19, *t* = 0.64), the relationship between the existence of a learning organization and organizational commitment was probably fully mediated by internal marketing [24]. To confirm the full mediation of internal marketing, a more parsimonious model without the direct paths was fitted. This model was then compared with Model I (Model II vs. Model I: Δχ^2^(1) = 0.31, *ns*). According to Hair et al. [22], a non-significant difference test indicates that the dropped pathway is not important to the model (Table 5). This reveals strong evidence for the mediation effect of internal marketing between the existence of a learning organization and organizational commitment.

**Table 5:** Fit indices for the structural equation models

**Model**	**χ**^**2 **^**( *df *)**	**△** ***χ***^**2**^	**AGFI**	**SRMR**	**CFI**	**RMSEA**
I	35.42 (17)	-	0.850	0.048	0.956	0.098
II	35.73 (18)	0.31	0.859	0.049	0.958	0.093

Note: Model I (with path c’) = model with direct pathways between LO and OC.

Model II (remove path c’) = model without direct pathways between LO and OC.

*χ*^*2*^ chi-square, *df* degrees of freedom, *AGFI* adjusted goodness of fit, *SRMR* standardized root-mean-squared residual, *CFI* comparative fit index, *RMSEA* root-mean-square error of approximation.

**Figure 1:**
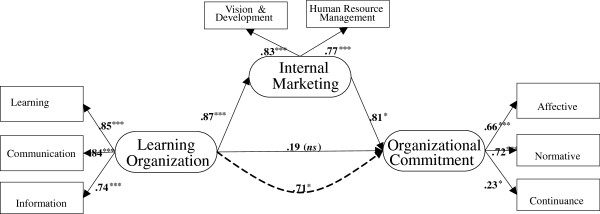
**The conceptual model of this study (Model I).** Note. AMOS standardized estimates for Model I. *p < .05, ***p < .001. Solid lines = direct effect, dashed line = indirect effect.

The conclusions drawn by the maximum likelihood and bootstrapping estimates were consistent (Table 4), suggesting that our procedure led to a stable estimate of the distributions (AMOS standardized estimates are provided in Figure 1).

## Discussion

We proposed four hypotheses:

Hypothesis 1- Creating a learning organization influences internal marketing.

Hypothesis 2- Internal marketing influences organizational commitment.

Hypothesis 3 - Creating a learning organization influences organizational commitment.

Hypothesis 4 - Internal marketing is a mediator between the existence of a learning organization and organizational commitment.

All four hypotheses were supported empirically. Estrada [25] studied acute care setting and found when nurses support evidence-based practice (EBP), it is helpful in promoting a learning organization culture. We found a statistically significant positive correlation between internal marketing and the existence of a learning organization. Through statistical analysis, we also found that internal marketing mediates between the existence of a learning organization and organizational commitment. That is, when hospitals promote a learning organization culture, internal marketing is helpful in positively influencing nurses’ organizational commitment. We therefore suggest that hospital managers should practise internal marketing to support the creation and ongoing existence of a learning organization.

The three constructs of ‘learning organization’ obtained from the study were learning, communication, and information. The learning construct had the highest average (3.64), followed by information (3.62), then communication (3.46). Snell and Hui [26] employed a case study approach to examine companies in China and Hong Kong. Using interviews, they tried to understand the major factors that influenced the success of the companies in promoting a learning organization culture. Their research found that communication was a very important factor. Watkins and Ellinger [13] also believed that managers of learning organizations should establish sound internal communication channels to promote mutual communications between managers and staff. In the learning organization items, we obtained the second lowest score (3.38) for “Staff can state their opinions and give feedback freely, regardless of their position”. In internal marketing and communication related items, “Our organization places significant emphasis on communication with employees” also received the lowest average (3.41). It is therefore suggested that communication channels between the nursing managers and staff should be strengthened to clearly convey the hospital’s vision and goals to frontline nurses.

We found that among all the learning organization items, “Administrators take long-term effects more seriously than short-term problem-solving” had the lowest average (3.36). Errors are not allowed in the process of healthcare since it affects patients’ lives and health; short-term healthcare problem may lead to long-term effect. The nursing staff competence in facing healthcare problems also affects patient care [27]. We therefore suggest that nursing managers should adopt a “double loop learning” [28] mentality and seek solutions to problems with a long-term perspective.

In our study, we found an item in organizational commitment, “It would take very little change in my present circumstances to cause me to leave this organization,” had the lowest average (3.20). Additionally, continuance commitment had the lowest average (3.21) among the three constructs of organization commitment, indicating that nurses have a low retaining intention in their hospitals. Organizational commitment can be used to predict nurses’ intent to leave [29], yet we found the respondent nurses had a low commitment to retaining their positions. This is an issue that warrants nursing managers’ attention. We also suggest that future studies may wish to explore the relationship between creation of a learning organization, employees’ organizational commitment and their turnover rate.

## Conclusions

Developing a learning organization emphasizes the fact that organizations must establish a work environment that provides and supports continual learning for employees [30]. To enhance nurses’ continual learning, knowledge updating, and professional competence, we suggest that nursing managers should practise internal marketing.

### Limitations and future research

The number of nurses willing to participate, and the fact that random sampling could not be conducted are possible limitations. Our respondents were nursing staff from only two hospitals in the city of Taichung, Taiwan. We intend to conduct further studies with an increased sample size to improve the potential for extrapolation of these results.

Our research revealed that of the three constructs of organizational commitment—affective, normative, and continuance commitment—the last had the lowest average. Additionally, continuance commitment and normative commitment also showed a low correlation coefficient (Table 3). This is probably one of the factors responsible for the low internal consistency of organizational commitment. Continuance commitment reflects an awareness on the part of nursing staff of the costs of leaving an organization, while normative commitment reflects an individual’s sense of obligation to remain in an organization. Future studies that investigate the factors that improve nursing staff organizational commitment should pay additional attention to continuance commitment, as this may be the factor causing low organizational commitment.

We also found a low correlation between a nurse’s realization of the cost of departure (i.e. valuable fringe benefits like bonuses) and whether they choose to stay in the organization from a sense of moral obligation. Jones and George [31] considered personal ethics and values to be influential on an employee’s attitude and conduct. The low correlation between these two constructs may be due to other antecedents that interfere with the organizational commitment of nursing staff, and the personal values/code of ethics relating to their conduct may be a predicator. Future studies that further explore the impact of values or code of ethics on organizational commitment may be useful.

## Competing interests

The author declares there are no competing interests for this manuscript.

## Authors’ contributions

YT participated in the sequence alignment and drafted the manuscript. YT participated in the design of the study and performed the statistical analysis. YT conceived of the study, and participated in its design and helped to draft the manuscript. The author read and approved the final manuscript.

## Pre-publication history

The pre-publication history for this paper can be accessed here:

http://www.biomedcentral.com/1472-6963/14/152/prepub

## Supplementary Material

Additional file 1: Table S1 Results of factor analysis for learning organizations. Click here for file

Additional file 2: Table S2 Results of factor analysis for internal marketing. Click here for file

Additional file 3: Table S3 Results of factor analysis for organization commitment. Click here for file
