# Supplementary material for: Learning organizations, internal marketing, and organizational commitment in hospitals
Source: BMC Health Serv Res. 2014 Apr 4;14:152. doi: 10.1186/1472-6963-14-152 (PMC4077678; doi:10.1186/1472-6963-14-152)
Supplement: Additional file 1: Table S1 — Results of factor analysis for learning organizations. [file 1472-6963-14-152-S1.doc]

Table S1. Results of factor analysis for learning organizations

|  |  |  | Factor loading | | |
| --- | --- | --- | --- | --- | --- |
| Constructs / content of items | Mean | SD | Factor 1 | Factor 2 | Factor 3 |
| ***Learning*** |  |  |  |  |  |
| •We look at innovation, unexpected incidents, and failure to be opportunities for learning in our hospital. | 3.78 | 0.63 | 0.78 | -0.08 | 0.28 |
| •The administrators provide sufficient resources to support learning activities in our hospital. | 3.50 | 0.72 | 0.71 | 0.31 | 0.02 |
| • The items in hospital performance evaluation include the staff members' learning effect. | 3.65 | 0.74 | 0.68 | 0.10 | 0.25 |
| •Learning pushes administrators to reform the organizational structure and redesign working processes. | 3.66 | 0.64 | 0.68 | 0.28 | 0.18 |
| •The rules of organization are set to encourage collective learning in our hospital. | 3.63 | 0.74 | 0.64 | 0.27 | 0.34 |
| •Self-criticism and action are both important in our hospital. | 3.63 | 0.76 | 0.64 | 0.56 | 0.04 |
| •The administrators encourage trying and examining methods that have been evaluated carefully in our hospital. | 3.55 | 0.73 | 0.62 | 0.29 | 0.33 |
| •Staff can learn from both processes and results in good job performance in our hospital. | 3.68 | 0.70 | 0.62 | 0.53 | -0.03 |
| •The administrators encourage two-way communication not only between individuals but also between departments in our hospital. | 3.64 | 0.69 | 0.59 | 0.16 | 0.35 |
| ***Communication*** |  |  |  |  |  |
| •Staff can state their opinions and give feedback freely, regardless of their position. | 3.38 | 0.78 | 0.12 | 0.80 | 0.22 |
| •Administrators take long-term effects more seriously than short-term problem-solving. | 3.36 | 0.81 | 0.16 | 0.70 | 0.20 |
| •Staff can challenge traditions in the pursuit of excellence. | 3.58 | 0.72 | 0.35 | 0.60 | 0.37 |
| •Respecting differences is part of the collective learning that is promoted in our hospital. | 3.57 | 0.80 | 0.36 | 0.56 | 0.42 |
| •Staffs are allowed to express their opinions because of the open rule. | 3.43 | 0.85 | 0.19 | 0.51 | 0.46 |
| ***Information*** |  |  |  |  |  |
| •Staff know how to acquire and apply these information appropriately. | 3.68 | 0.80 | 0.30 | 0.01 | 0.81 |
| •We will collect and consider useful information from customers and suppliers. | 3.62 | 0.70 | 0.15 | 0.29 | 0.75 |
| •Staff can seek information not included in their professional domain. | 3.61 | 0.69 | 0.12 | 0.35 | 0.59 |
| •Policies and rules are set to make learning easy for the staff. | 3.56 | 0.74 | 0.40 | 0.40 | 0.59 |
| Variance explained (%) |  |  | 25.53 | 18.83 | 16.97 |
| Cronbach’s α |  |  | 0.90 | 0.83 | 0.81 |
