# Supplementary material for: Learning organizations, internal marketing, and organizational commitment in hospitals
Source: BMC Health Serv Res. 2014 Apr 4;14:152. doi: 10.1186/1472-6963-14-152 (PMC4077678; doi:10.1186/1472-6963-14-152)
Supplement: Additional file 2: Table S2 — Results of factor analysis for internal marketing. [file 1472-6963-14-152-S2.doc]

Table S2. Results of factor analysis for internal marketing

|  |  |  | Factor loading | |
| --- | --- | --- | --- | --- |
| Constructs / content of items | Mean | SD | Factor 1 | Factor 2 |
| ***Vision & Development*** |  |  |  |  |
| •Our organization offers employees a vision that they can believe in. | 3.42 | 0.69 | 0.80 | 0.15 |
| •The vision of our organization is well communicated to all employees. | 3.49 | 0.73 | 0.79 | 0.19 |
| •Our organization views the development of knowledge and skills in employees as an investment rather than a cost. | 3.42 | 0.74 | 0.78 | 0.26 |
| • Our organization makes preparations for employees to perform well. | 3.43 | 0.66 | 0.72 | 0.27 |
| • Our organization teaches employees "why they should do things" and not just "how they should do things." | 3.47 | 0.72 | 0.72 | 0.18 |
| •The development of employee skills and knowledge is an ongoing process in our organization. | 3.54 | 0.71 | 0.69 | 0.38 |
| •Our organization goes beyond simple training and educates employees to work together. | 3.54 | 0.75 | 0.56 | 0.36 |
| •Our organization measures and rewards employee performance that contributes to achieving the organizational vision. | 3.50 | 0.74 | 0.41 | 0.40 |
| ***Human Resource Management*** |  |  |  |  |
| •In our organization, employees are properly trained to perform their service roles. | 3.54 | 0.75 | 0.07 | 0.82 |
| •Data gathered from employees are used to improve jobs and develop organizational strategies. | 3.55 | 0.77 | 0.29 | 0.79 |
| •In our organization, employees who provide excellent service are rewarded for their efforts. | 3.45 | 0.79 | 0.30 | 0.73 |
| •Our organization communicates to employees the importance of their service roles. | 3.54 | 0.67 | 0.35 | 0.72 |
| •Our organization places significant emphasis on communication with employees. | 3.41 | 0.76 | 0.19 | 0.71 |
| •Our organization has the flexibility to accommodate different employee needs. | 3.45 | 0.75 | .32 | .70 |
| Variance explained (%) |  |  | 30.88 | 28.57 |
| Cronbach’s α |  |  | 0.88 | 0.88 |
