# Supplementary material for: Learning organizations, internal marketing, and organizational commitment in hospitals
Source: BMC Health Serv Res. 2014 Apr 4;14:152. doi: 10.1186/1472-6963-14-152 (PMC4077678; doi:10.1186/1472-6963-14-152)
Supplement: Additional file 3: Table S3 — Results of factor analysis for organization commitment. [file 1472-6963-14-152-S3.doc]

Table S3. Results of factor analysis for organization commitment

|  |  |  | Factor loading | | |
| --- | --- | --- | --- | --- | --- |
| Constructs / content of items | Mean | SD | Factor 1 | Factor 2 | Factor 3 |
| ***Affective Commitment*** |  |  |  |  |  |
| •I find that my values and the organization's values are very similar. | 3.33 | 0.76 | 0.78 | 0.11 | 0.04 |
| •I am willing to put in a great deal of effort beyond that normally expected to help this organization become successful. | 3.50 | 0.73 | 0.76 | 0.23 | -0.02 |
| •I would accept almost any type of job assignment to keep working for this organization. | 3.29 | 0.76 | 0.72 | 0.04 | 0.17 |
| •I talk about this organization with my friends and tell them it is a great organization to work for. | 3.45 | 0.77 | 0.64 | 0.22 | -0.15 |
| •I am proud to tell others that I am part of this organization. | 3.38 | 0.72 | 0.55 | 0.48 | -0.06 |
| ***Normative Commitment*** |  |  |  |  |  |
| •I really care about the fate of this organization. | 3.61 | 0.79 | 0.08 | 0.74 | -0.03 |
| •For me, this is the best of all possible organizations. | 3.24 | 0.79 | 0.17 | 0.71 | 0.03 |
| •I am extremely glad that I chose this organization to work for over others I was considering at the time I joined. | 3.55 | 0.74 | 0.15 | 0.63 | 0.38 |
| •There is not too much to be gained by sticking with this organization. | 3.45 | 0.77 | 0.33 | 0.56 | 0.19 |
| ***Continuance Commitment*** |  |  |  |  |  |
| •It would take very little change in my present circumstances to cause me to leave this organization. | 3.20 | 0.82 | 0.19 | -0.17 | 0.83 |
| •I do not believe that I will have a future if I keep working in this hospital. | 3.22 | 0.73 | 0.01 | 0.16 | 0.83 |
| •Often, I find it difficult to agree with this organization's policies on important matters related to its employees. | 3.22 | 0.78 | -0.24 | 0.29 | 0.78 |
| Variance explained (%) |  |  | 22.31 | 18.81 | 18.58 |
| Cronbach’s α |  |  | 0.78 | 0.69 | 0.77 |
